# Advances on the Failure Analysis of the Dam—Foundation Interface of Concrete Dams

**DOI:** 10.3390/ma8125442

**Published:** 2015-12-02

**Authors:** Luis Altarejos-García, Ignacio Escuder-Bueno, Adrián Morales-Torres

**Affiliations:** 1 Universidad Politécnica de Cartagena, Ud. Predepartamental de Ingeniería Civil, Paseo de Alfonso XIII, 52, Cartagena 30203, Spain; luis.altarejos@upct.es; 2 Instituto de Ingeniería del Agua y Medio Ambiente, Universitat Politècnica de València, Camino de Vera SN. Valencia 46022, Spain; 3 iPresas Risk Analysis, Av. Del Puerto N.180. 1-B., Valencia 46023, Spain

**Keywords:** concrete gravity dams, sliding failure mode, reliability, uncertainty, spatial variability

## Abstract

Failure analysis of the dam-foundation interface in concrete dams is characterized by complexity, uncertainties on models and parameters, and a strong non-linear softening behavior. In practice, these uncertainties are dealt with a well-structured mixture of experience, best practices and prudent, conservative design approaches based on the safety factor concept. Yet, a sound, deep knowledge of some aspects of this failure mode remain unveiled, as they have been offset in practical applications by the use of this conservative approach. In this paper we show a strategy to analyse this failure mode under a reliability-based approach. The proposed methodology of analysis integrates epistemic uncertainty on spatial variability of strength parameters and data from dam monitoring. The purpose is to produce meaningful and useful information regarding the probability of occurrence of this failure mode that can be incorporated in risk-informed dam safety reviews. In addition, relationships between probability of failure and factors of safety are obtained. This research is supported by a more than a decade of intensive professional practice on real world cases and its final purpose is to bring some clarity, guidance and to contribute to the improvement of current knowledge and best practices on such an important dam safety concern.

## 1. Introduction

When failures of complex structures are analyzed, evaluation of uncertainty should play an important role in the analysis of the behavior of a constructed facility. In general, two sources of uncertainty should be considered [1,2]. Natural uncertainty or randomness is produced by the inherent variability of natural processes. An example of this kind of uncertainty is the variability of the loads a structure has to withstand, for instance, the variability in the earthquakes intensity that can occur. Another example is the resistance’s variability of the terrain where the structure is settled. This type of uncertainty, sometimes also called aleatory uncertainty, cannot be reduced, though it can be estimated [3,4]. Epistemic uncertainty results from not having enough knowledge or information about the analyzed system. This lack of information can be due to a data deficiency or an inappropriate representation of the structure’s behavior. The more knowledge is available about a structure, the more this type of uncertainty can be reduced. On the other hand, it is usually very difficult to estimate or quantify this uncertainty. An example of this type of uncertainty can also be found in the resistance of the terrain. The information about the foundations may be limited so the parameters used to characterize its resistance are estimated from probing and exploration. With more resources, foundations can be better characterized and the epistemic uncertainty reduced, although the natural variability may still be very significant.

The distinction between natural and epistemic uncertainty becomes more important for a quantitative risk analysis in complex structures. In this context, natural uncertainty is usually related to the occurrence of events able to produce the structural failure and the randomness of the structure’s resistant behavior for the load produced by the events. In contrast, epistemic uncertainty is mainly focused on the lack of knowledge about the failure mechanisms, the structure’s resistance parameters and the consequences produced by the failure.

In any case, characterization of uncertainty is an inherent process in any risk analysis, since it encompasses the failure probability concept. If there were no uncertainties, the load situation that produces the structural failure with 100% probability could be determined with precision.

The failure mode considering the sliding of a concrete gravity dam along the dam-foundation contact is one of the most important safety issues in dam engineering. It is included in all dam design codes and safety guidelines and standards known to the authors across different countries [5]. The approach followed to ensure that an acceptable level of safety is achieved in the design stage is based on a combination of experience, a conservative approach to models and a prudent estimate of shear strength properties in the dam-foundation contact. Once the dam is in operation, periodical safety reviews are conducted to evaluate dam condition and to check if there have been any changes that may affect the safety level.

Some factors which may contribute to impair the desired safety level of a concrete dam are:

- Water levels in the reservoir higher than design water levels;
- Uplift pressures higher than design;
- Lack of knowledge on shear strength parameters along the failure surface;
- Degradation/aging phenomena in the dam-foundation contact, with loss of shear strength.

Behind the higher than design water levels in the reservoir, one can find aspects such as the increase in flood magnitude and frequency due to climate change and gate failures during flood routing operations [6,7]. Uplift pressures can be partially known, and only to the extent that a monitoring system is implemented and properly managed, and pressures over the maximum design have been reported in some dams [8,9]. Lack of knowledge on shear strength parameters is another common issue in dam safety reviews. Shear strength parameters are often estimated based on few on-site specific data. Degradation phenomena in dam-foundation contacts have been also reported in a number of cases [10,11] thus needing for remedial actions.

It is clear from this picture that uncertainty is playing a key role in the assessment of the safety of an existing concrete dam, whose failure may cause high damages in terms of human lives, the environment and infrastructures in the downstream areas. This combination of uncertainty and potential high damages has made attractive the use of a risk based approach to the problem of the safety assessment of existing dams [12]. Though risk can be defined in a number of different ways [13] in the context of dam safety risk is defined as the product of the probability of failure times the consequences of failure [14].

The analysis of the response of a structural system to a certain loading scenario in terms of probability of failure has been addressed using the reliability theory, which is based on the theories of probability and statistics. This approach has been successfully used along different sectors such as the nuclear, chemical or aeronautical, where probability of failure can be interpreted in terms of relative failure frequencies observed form operational experience. In civil engineering structures this approach encounters difficulties, as failures are events with low frequency of occurrence. In addition, safety of civil engineering structures is closely related with the foundation, so we are dealing with prototypes with unique features [15].

The estimation of the probability of failure of the dam is one of the critical aspects of a risk based approach, and a lot of attention has been drawn to its meaning and to search ways to consistently derive probability values that can be used in a risk analysis [16]. Dam safety practitioners and researchers have addressed issues such as the conceptual, scientific validity of the approach [17], reliability concepts [18] and models. The authors’ experience, based on 10 years of real world practice of risk analysis applied to more than 20 concrete gravity dams across the world, is that part of the profession, including dam owners, engineers and consultants, has serious objections regarding the use and validity of probabilities of failure for safety assessments of concrete dams. This fact is usually identify or termed as a “gap” between engineering practice and engineering research.

In this paper, our objective is to contribute to bridge that gap, offering some thoughts on the analysis of the probability of sliding of a concrete dam along its base, that, according to our understanding, may help to clarify concepts and to contribute to the improvement of the current knowledge and best practices. To illustrate the reasoning, we want to follow in this paper we will use a case study described in the following sections.

We will show four different but complementary approaches to the analysis of the sliding problem under a reliability perspective, taking into account the distinction between spatial variability of local strength parameters and spatial variability of local strength itself along the failure surface, and how local values relate to average values over the whole dam-foundation contact. These four approaches are identified as:

- Approach I—Parameter average with no data;
- Approach II—Parameter average with data;
- Approach III—Parameter average with variance reduction factor;
- Approach IV—Spatially variable strength.

The first approach considers average values of strength parameters over the failure surface under the hypothesis of lack of on-site specific data, thus allowing for high levels of uncertainty on the probability distributions of such average parameters.

The second approach uses also average values of strength parameters but shows how uncertainty is reduced as the standard deviation of probability distributions of strength parameters diminishes when more information is incorporated into the analysis. These two approaches to the analysis of the sliding problem under a reliability perspective are commonly used in risk analysis practice, and none of them takes into account the effect of spatial variability of strength parameters and strength forces.

The third approach considers average values of strength parameters, but takes into account the spatial variability of strength parameters and its relation to the extension of the failure surface using the variance reduction factor [19].

The fourth approach does not consider average values of strength parameters, but evaluates shear strength locally taking into account parameter variability and the spatial variability of other influencing factors, such as the normal effective stress over the failure surface.

The paper is organized as follows. The sliding strength model and the associated uncertainties, which are important to inform modelling decisions, are discussed in Section 2. The above mentioned four approaches are described in detail in Section 3. The case study dam and the results obtained in terms of safety factors and probabilities of failure are presented in Section 4. The discussion of results is included in Section 5 whereas conclusions are shown in Section 6.

## 2. Sliding Strength Models and Uncertainties

### 2.1. The Mohr-Coulomb Strength Model

The most extended model used in practical applications to analyze the sliding strength of the dam-foundation contact is the Mohr-Coulomb model. In this section, we will discuss the uncertainties present in the Mohr-Coulomb model.

For a monolith of a concrete dam, the model predicts sliding strength, *R*, by Equation (1), where *N* is the sum of normal forces to dam-foundation contact plane, *U* is the sum of pore pressure forces acting on the contact plane, *S* is the contact area, $\bar{\text{tan}\varphi }$ is the average friction coefficient over the contact plane and $\bar{coh}$ is the average cohesion over the contact plane.

(1) $$R=\left(N-U\right)\bar{\text{tan}\varphi }+S\cdot \bar{coh}$$

This rigid body model assumes the Navier hypothesis and postulates linear normal stress distributions in the dam foundation contact. The model is refined with the following considerations:

- Cohesion acts only on zones subjected to compressive effective stresses;
- If normal effective tensile stresses exceeds the tensile strength of the contact, a crack will develop, starting at the upstream end. The value of uplift inside the crack will be constant and equal to the value of uplift at the upstream end.

The model expresses the sliding strength as a function of strength parameters, drainage effectiveness and water level in the reservoir. This model can be seen as a generalization for the whole sliding plane of the local sliding strength at each point of the surface that is described by Equation (2), where σ_n_ is the total normal stress, u is the pore pressure, tanφ is the local friction coefficient and *coh* is the local cohesion.

(2) $$\tau =\left(\sigma_{n}-u\right)\text{tan}\varphi +coh$$

If we denote by Ω the whole contact plane, then we can establish the relationship between local strength and total strength, by Equation (3).

(3) $$R=\underset{\Omega }{\int }\tau \text{ d}\Omega$$

If we denote by *L* the sum of sliding forces acting in a direction parallel to the sliding surface, the factor of safety is defined by Equation (4).

(4) $$FS=\frac{R}{L}$$

It should be stressed that the model thus defined is based not only on geotechnical parameters, but also reflects the impact of operational issues, as the uplift pressure is directly related to drainage system implementation, performance, monitoring and maintenance.

One characteristic of this model is that it exhibits a strong softening behaviour, as the sliding strength is reduced with increasing loading levels. This behavior is due to two effects:

- As water level rises, the uplift increases, reducing frictional strength;
- As water level rises, due to the increase in the overturning moment, tensile stresses may develop at the upstream toe leading to cracking and therefore reducing the area of the contact plane under compressive stresses. This fact has two consequences: loss of cohesive strength and increase in shear stresses, which may reach the peak shear strength locally.

**Figure 1:**
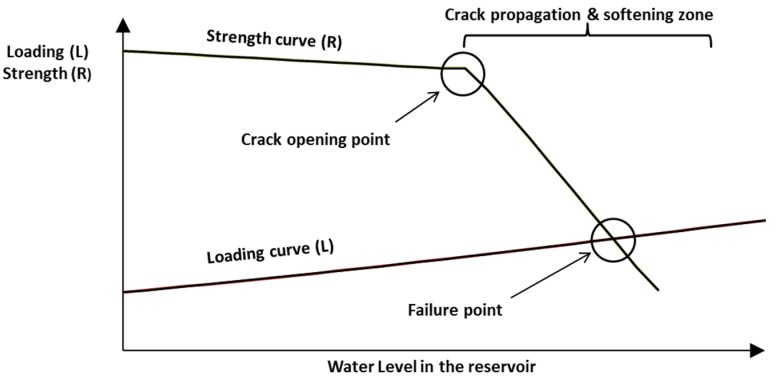
Reduction of sliding strength with pool levels.

Figure 1 illustrates this softening behavior. Before cracking occurs, the strength reduction is smooth. After the crack develops, higher water levels will lead to a steep, fast reduction of strength reserve.

### 2.2. Uncertainties in the Strength Model

The first source of uncertainty is associated with the selection of the Mohr-Coulomb model itself instead of other sliding models. The strength defined in Equation (2) can be implemented in a discrete limit equilibrium or in a numerical model. In this latter case, together with the description of the shear stress-shear strain curve, it can be used to simulate the sliding process in a more realistic way [20]. This approach allows to incorporate a more precise, non-linear normal stress distribution. It also enables the use of spatially distributed values of sliding strength parameters, tanφ and *coh*. The practical difficulty of the application comes from the fact that the local values of strength parameters over the whole sliding plane are not known. The engineer only has information regarding a few values, if any, over the entire sliding surface. In addition, numerical models are time and resource consuming in a risk analysis context, so in common practice, limit equilibrium models with single values of strength parameters are typically used.

There are some key aspects to consider if a Mohr-Coulomb model is to be used to derive probabilities of failure in a reliability analysis. First of all, the average values of strength parameters are usually not known with precision. If a reliability analysis is to be done, probability distributions and their parameters should be defined, which is a task subjected to a strong subjective judgement from the modeler.

Data from tests refer to local values and not to the average values needed by the model. One source of uncertainty is the inherent, natural variability of the strength parameters, which is reflected through the dispersion of values observed in the test results performed at dam sites. This variability is essentially spatial variability, which is seldom fully accounted in practical applications, as the objective is typically to get a prudent estimate of the average values of strength parameters. In this paper we are focused on this type of uncertainty.

Another source of uncertainty refers to the distinction between bonded and unbonded zones in the contact area. Tests carried out by Lo [21] for cores from several dams in Canada showed that the contact may appear unbonded or weakly bonded. Regarding the available information on geology, dam construction, water seepage and filtrations, the team performing the analysis has to assess the possibility of having unbonded areas in the dam-foundation contact. In bonded zones, the shear strength is characterized by higher friction coefficient and cohesion values, close to peak values. In unbonded zones residual values should be used. In these unbonded zones, cohesion is null and friction coefficients are typically lower, close to those corresponding to basic friction angles. We will not include this sort of uncertainty, as the discussion of its extension and spatial distribution is out of the scope of the paper, though from an operational perspective it can be easily included in the methodological approaches proposed.

An additional source of uncertainty is related to tensile strength in the dam-foundation contact. It has been shown [21] that samples retrieved from concrete-rock contacts exhibit significant tensile strength. A relationship between cohesion and tensile strength based on Griffith’s criteria can be used [22]. The tensile strength controls the opening and propagation process locally when tensile stresses appear at the upstream heel of the dam. To simplify the analysis, we will consider null tensile strength for the case study dam in this paper, though the impact of this parameter on dam safety using the models presented is currently under research by the authors. In addition, weak planes in the rock foundation below the dam might be more critical, which supports this assumption.

Apart from this purely geotechnical aspects, there are two other key sources of uncertainty: drainage system effectiveness and water levels in the reservoir. Three situations are usually considered for drains: fully effective, partially effective and fully ineffective. Changes in uplift values have major consequences on dam stability, so it is a good practice to devote enough attention to the sound assessment of drain performance. The probabilities assigned to each situation will reflect the team opinion on the maintenance and monitoring practices followed by the dam owner. The other source of uncertainty is the water level in the reservoir. The reservoir level is controlled by bottom outlet works and gated spillways. The valves and gates performance in terms of reliability and the decision making processes of outlets and gates operations based on operational rules and flood forecasting play a key role in the determination of the maximum water level during a flood event.

## 3. Modeling Approaches

It has been shown that uncertainty spreads all over the sliding stability model, including geotechnical aspects related with strength parameters, but also operational, maintenance, monitoring and inspection issues related with drainage system performance and water level in the reservoir. In this context, risk analysis is an attractive option to deal with safety issues. Risk analysis uses the concept of probability as a measure of uncertainty, so reliability-based approaches are incorporated into risk analysis.

We will consider four approaches in the analysis of the sliding problem under a reliability perspective, focusing on the distinction between spatial variability of local strength parameters and spatial variability of local strength itself along the failure surface. These four approaches are:

- Approach I—Parameter average with no data;
- Approach II—Parameter average with data;
- Approach III—Parameter average with variance reduction factor;
- Approach IV—Spatially variable strength.

### 3.1. Approach I—Parameter Average with No Data

The first approach considers average values for strength parameters over the failure surface under the hypothesis of lack of on-site specific data, thus allowing for high levels of uncertainty on the probability distributions of such average parameters. This approach assumes average values of friction coefficient, tanφ_contact_, and cohesion, *coh*_contact_, for the whole contact plane. In the absence of specific, on-site data, characteristic values of strength parameters are proposed by the engineer based on his/her own experience or published data of dams with similar characteristics and geology features. Then, a factor of safety against sliding is calculated. The characteristic values shall be selected as cautious estimates of the value affecting the occurrence of the limit state, thus the characteristic value is normally a prudent estimate of the average value on the failure surface, not a particular fractile of the test results. According to Eurocode 7 [23], the calculated probability of a worse value governing the occurrence of the limit state under consideration should not be greater than 5% [24].

Under a reliability perspective, we are interested in the assessment of the probability associated with such average strength parameters. Assuming probabilistic normal distributions and independence, we have Equations (5) and (6). The use of normal distributions has its limitations derived from the unbonded tails and possibility of negative values without physical meaning. Nevertheless, for the sake of simplicity and to keep the focus on the comparison of different approaches and not on the discussion of probability distributions the normal probability distribution is used.

(5) tanφ_contact_ ~ N(μ[tanφ_contact_]; SD[tanφ_contact_])

(6) *coh*_contact_ ~ N(μ[*coh*_contact_]; SD[*coh*_contact_])

where μ[tanφ_contact_] is the mean and SD[tanφ_contact_] is the standard deviation of the friction coefficient for the whole contact plane, while μ[*coh*_contact_] is the mean and SD[*coh*_contact_] is the standard deviation of cohesion for the whole contact plane.

It is assumed that at any point on the failure surface, the local strength parameters are realizations of a random field where μ[tanφ_local_] is the mean and SD[tanφ_local_] is the standard deviation of local friction coefficient, while μ[*coh*_local_] is the mean and SD[*coh*_local_]) is the standard deviation of local cohesion. These probabilistic parameters are the same at each point of the failure surface. If probabilistic normal distributions are assumed for local friction coefficient, tanφ_local_, and for local cohesion, *coh*_local_, and assuming that they are statistically independent, uncorrelated random values, then we have Equations (7) and (8).

(7) tanφ_local_ ~ N(μ[tanφ_local_]; SD[tanφ_local_])

(8) *coh*_local_ ~ N(μ[*coh*_local_]; SD[*coh*_local_])

In the absence of information on spatial variability, which can be expressed in the form of correlation distances or scale of fluctuation, the approach followed in this case of no information is to let the average values to vary over the full range of variation of the local strength parameters, as shown in Equations (9)–(12).

(9) μ[tanφ_contact_] = μ[tanφ_local_]

(10) SD[tanφ_contact_] = SD[tanφ_local_]

(11) μ[*coh*_contact_] = μ[*coh*_local_]

(12) SD[*coh*_contact_] = SD[*coh*_local_]

Conceptually, this approach assumes that the average strength parameters for the whole contact plane may take any value randomly extracted from the full range of variation of the local strength parameters. This approach assumes maximum uncertainty on strength values, and it is equivalent to consider an infinite scale of fluctuation of strength parameters in relation to the extension of the failure surface. The calculation of the factor of safety is carried out selecting a cautious estimate of the value of the corresponding strength parameter. Following Eurocode 7 [23] and in the absence of data we will use the 5% fractile as the characteristic value.

The calculation of the probability of failure is carried out using the full probability distributions defined in Equations (5) and (6), with a Monte Carlo based methodology [25] that incorporates the limit state curve concept.

### 3.2. Approach II—Parameter Average with Data

The second approach uses also average values for strength parameters over the failure surface, but incorporates a reduction on uncertainty as more information is added to the analysis. Under a reliability perspective, we accept that data of local strength values represent realizations of random values from their independent, uncorrelated normal probability distributions, according to Equations (7) and (8). We are assuming that local strength parameters vary spatially over the whole contact plane so they can be described by Gaussian random fields. In this context, strength data are understood as realizations of Gaussian random fields, so they are normally distributed.

We have a number of observations, *n*, of friction coefficient and cohesion. The mean of the observed values for both parameters is, respectively, $\bar{\text{tan}\varphi }$ and $\bar{coh}$. The mean of the observed values is an estimator of the expected value of the population and it is normally distributed, with parameters given by Equations (13)–(16).

(13) $$\mu \left[\bar{\text{tan}\;\varphi }\right]=\mu \left[{\text{tan}\varphi }_{\text{local}}\right]$$

(14) $$\text{SD}\left[\bar{\text{tan}\;\varphi }\right]=\frac{\text{SD}\left[{\text{tan}\varphi }_{\text{local}}\right]}{\sqrt{n}}$$

(15) $$\mu \left[\bar{coh}\right]=\mu \left[coh_{local}\right]$$

(16) $$\text{SD}\left[\bar{coh}\right]=\frac{\text{SD}\left[coh_{local}\right]}{\sqrt{n}}$$

Without other considerations, a first approximation relies on evaluating average strength parameter values in the contact by the mean values obtained with the n data, as shown in Equations (17)–(20).

(17) $$\mu \left[{\text{tan}\varphi }_{\text{contact}}\right]=\mu \left[\bar{\text{tan }\varphi }\right]$$

(18) $$\text{SD}\left[{\text{tan}\varphi }_{\text{contact}}\right]=\text{SD}\left[\bar{\text{tan }\varphi }\right]$$

(19) $$\mu \left[coh_{\text{contact}}\right]=\mu \left[\bar{coh}\right]$$

(20) $$\text{SD}\left[coh_{\text{contact}}\right]=\text{SD}\left[\bar{coh}\right]$$

An important underlying assumption needed is to accept that the on-site data are evenly distributed over the contact plane, being representative of different zones of similar extension, so we can express the probability distribution of average strength parameter values using the probability parameters of the n local strength values retrieved from tests. The expected value of the average is the mean of the probability distribution of local parameters and that the standard deviation of the average is reduced with respect the standard deviation of local parameters. Conceptually, this approach assumes that the average strength parameters for the whole contact plane are estimated using local strength parameters, and as more information is gained less room is left to variability. We will use the 5% fractile as the characteristic value to calculate the factor of safety. The calculation of the probability of failure is made in the same fashion described for Approach I.

Approaches I and II applied to the analysis of the sliding problem under a reliability perspective are commonly used in risk analysis practice, and none of them takes into account the effect of spatial variability of strength parameters and strength forces.

### 3.3. Parameter Average with Variance Reduction Factor

The third approach considers also average values for strength parameters, but takes into account their spatial variability and relation to the extension of the failure surface using the variance reduction factor. One way to account for the spatial variability is using the approach proposed by Phoon and Kulhawy [26], expressed by Equation (21).

(21) $$COV_{\text{TOTAL}}=\sqrt{\Gamma_{\text{S}}^{2}COV_{\text{inher}}^{2}+COV_{\text{meas}}^{2}+COV_{\text{trans}}^{2}+COV_{\text{stat}}^{2}}$$

where Г_S_^2^ is the variance reduction factor, considering the spatial extent of the governing failure mechanism; *COV_inher_* is the coefficient of variation of the parameter inherent variability; *COV*_meas_ is the coefficient of variation of the measurement errors; *COV*_trans_ is the coefficient of variation of the transformation errors; and *COV*_stat_ is the coefficient of variation of the statistical parameters.

The variance reduction function can be approximated by Equation (22) as proposed by Schneider and Schneider [27].

(22) $$\Gamma_{\text{S}}^{2}=\Gamma_{X}^{2}\Gamma_{Y}^{2}$$

where Г*_X_*^2^ is the variance reduction factor in the horizontal direction *X*, which is the upstream-downstream direction, and Г_Y_^2^ is the variance reduction factor in the horizontal direction *Y*, which the left-right direction of the failure surface. The variance reduction factor in a particular direction, *i*, is calculated using Equations (23) and (24) which represent a simplification of Vanmarcke’s Equations [28].

(23) $$\Gamma_{\text{i}}^{2}=\frac{SOF_{\text{i}}}{L_{\text{i}}}\left(1-\frac{SOF_{\text{i}}}{3L_{\text{i}}}\right)$$

(24) $$\Gamma_{\text{i}}^{2}=\left(1-\frac{L_{\text{i}}}{3\;SOF_{\text{i}}}\right)$$

where *SOF_i_* is the Scale of Fluctuation of the strength parameter in the direction i and *L*_i_ is the extent of the failure mechanism in the direction i. If *SOF_i_* > *L*_i_ Equation (23) is used. If *L*_i_ ≤ *SOF_i_* then Equation (24) is used.

Assuming best standard practices in the testing procedure, then *COV*_meas_ ≅ 0. If a well-established model is used to transform measured test results into the required parameter, then *COV*_trans_ ≅ 0. Assuming that the probabilistic parameters that describe the statistical distribution are known, then *COV*_stat_ ≅ 0.

Once the coefficient of variation has been estimated, the calculation of the new total standard deviation of strength parameters is straightforward, and the parameters of the new normal probability distributions are defined. We will use the 5% fractile as the characteristic value to calculate the factor of safety. The calculation of the probability of failure follows the same procedure as with Approaches I and II.

### 3.4. Spatially Variable Strength

The fourth approach does not consider average values for strength parameters, but evaluates shear strength locally taking into account parameter variability and the spatial variability of other influencing factors, such as the normal effective stress variability over the failure surface, and then sums local strengths to evaluate total strength. In this case, we assume that the local strength parameters are independent, uncorrelated random variables with spatial variation represented by random fields.

We consider the failure surface of length, *L*, and width, *W*, as a regular grid of I rows which follow the left-right direction and J columns in the upstream-downstream direction, dividing the surface into *I* × *J* = *M* cells. Each cell is a square, so *W*/*J* = *L*/*I*, and has an area s(i,j) = *S*/*M*, where S is the total area of the failure surface. The tensile strength that can be sustained by each cell, τ(i,j), is given by Equation (25).

(25) $$\tau \left(\text{i},\text{j}\right)={\sigma \prime }_{\text{n}}\left(\text{i},\text{j}\right)\cdot \text{tan}\varphi \left(\text{i},\text{j}\right)\cdot \delta \left(\text{i},\text{j}\right)+coh\left(\text{i},\text{j}\right)\cdot \delta \left(\text{i},\text{j}\right)$$

where σ^′^_n_(i,j) is the average normal effective stress acting on cell (i,j), tanφ(i,j) is the friction coefficient of cell (i,j), *coh*(i,j) is the cohesion of cell (i,j) and δ(i,j) is the indicator function defined by Equation (26).

(26) $$\delta \left(\text{i},\text{j}\right)=1;{\text{ if }\sigma^{\prime }}_{\text{n}}\left(\text{i},\text{j}\right)>0\;\delta \left(\text{i},\text{j}\right)=0;{\text{ if }\sigma^{\prime }}_{\text{n}}\left(\text{i},\text{j}\right)\leq 0$$

The sliding strength of each cell, r(i,j) is defined according to Equation (27).

(27) r(i,j)=τ(i,j)⋅s(i,j)

And the total sliding strength along the failure surface, *R_S_*, is given by Equation (28).

(28) $$R_{S}=\overset{\text{I}}{\underset{\text{i}=1}{\sum }}\overset{\text{J}}{\underset{\text{j}=1}{\sum }}\text{r}\left(\text{i},\text{j}\right)$$

As *R_S_* is the sum of M random variables normally distributed, it will be also normally distributed. Under the hypothesis of no spatial correlation in strength parameters, which means a null scale of fluctuation, the expected value, *E*[*R_S_*]*,* and the variance, *VAR*[*R*_S_], can be estimated by Equations (29) and (30).

(29) $$E\left[R_{\text{S}}\right]=\overset{\text{I}}{\underset{\text{i}=1}{\sum }}\overset{\text{J}}{\underset{\text{j}=1}{\sum }}{\sigma^{\prime }}_{\text{n}}\left(\text{i},\text{j}\right)\cdot \mu \left[\text{tan}\varphi \right]\cdot \text{s}\left(\text{i},\text{j}\right)\cdot \delta \left(\text{i},\text{j}\right)\;+\overset{\text{I}}{\underset{\text{i}=1}{\sum }}\overset{\text{J}}{\underset{\text{j}=1}{\sum }}\mu \left[coh\right]\cdot \text{s}\left(\text{i},\text{j}\right)\cdot \delta \left(\text{i},\text{j}\right)$$

(30) $$VAR\left[R_{\text{S}}\right]=\overset{\text{I}}{\underset{\text{i}=1}{\sum }}\overset{\text{J}}{\underset{\text{j}=1}{\sum }}{{\sigma^{\prime }}_{\text{n}}}^{2}\left(\text{i},\text{j}\right)\cdot VAR\left[\text{tan}\varphi \right]\cdot {\text{s}}^{2}\left(\text{i},\text{j}\right)\cdot \delta^{2}\left(\text{i},\text{j}\right)\;+\overset{\text{I}}{\underset{\text{i}=1}{\sum }}\overset{\text{J}}{\underset{\text{j}=1}{\sum }}VAR\left[coh\right]\cdot {\text{s}}^{2}\left(\text{i},\text{j}\right)\cdot \delta^{2}\left(\text{i},\text{j}\right)$$

With the factor of safety defined by Equation (31) and the probability of failure, *P*_fail_, defined by Equation (32), we can use the normal probability distribution of *R_S_* to estimate the probability of failure.

(31) $$FS=\frac{R_{S}}{L}$$

(32) $$P_{\text{fail}}=P\text{rob}\left[FS<1\right]=P\text{rob}\left[R_{\text{S}}-L\right]$$

If there is spatial correlation in strength parameters, the Equations (29) and (30) cannot be applied, and a different strategy is needed. If we generate a set of *i* = 1, 2,.., N realizations of the random fields with a certain scale of fluctuation, SOF, for both coefficient of friction and cohesion, we will get a set of different N values of *R*_S_, and, in accordance, a set of different N values of the factor of safety, which is defined by Equation (33).

(33) $$FS_{\text{i}}=\frac{{R_{\text{S}}}_{\text{i}}}{L}$$

If a large number of random fields, *N*, is generated, then the probability of failure can be estimated as the ratio between the number of sets where *FS*_i_ < 1, *N*_f_, and the total number of sets, *N*. Obviously, to be able to capture low orders of magnitude of the probability of failure, a large value of N is needed. Another strategy to approximate the probability of failure is to calculate a shorter number of random fields, retrieve the *FS*_i_ values, and calculate the expected value and the standard deviation of the sample of *FS*. Then, assuming that *FS* is normally distributed, the probability of failure can be calculated according to Equation (32). This latter approach is followed in this paper.

If we make the hypothesis that the distance of correlation of random fields is zero or close to zero, we are accepting that low local values will be compensated with higher local values occurring in the vicinity. If we consider a significant positive spatial correlation we get a different picture. Under this hypothesis of positive correlation distance, the cohesive part of the sliding strength still compensates lower values in some zones with higher values in other zones, but the frictional part of the sliding strength no longer exhibits such compensation effect. This is due to the asymmetrical distribution of compressive normal effective stresses along the contact plane. For typical pool water levels in the reservoir close to the maximum normal operation level or for higher levels under flood conditions the compressive normal effective stresses are higher at the downstream part of the contact plane and lower at the upstream part. Under some circumstances, even tensile effective stresses may develop at the upstream heel leading to the opening and propagation of a crack, changing the contact area under compressive stress even further.

A reliability analysis based on probability distributions of overall contact strength parameters cannot be carried out rigorously, because local effects and asymmetrical distribution of local strengths become important. A practical problem arises under this approach, which is the estimation of the correlation distance for each strength parameter. The assessment of this correlation distance or scale of fluctuation can only be gained with an extensive testing program, usually out of the scope of typical dam safety reviews.

However, the effect of the different scales of fluctuation over the factor of safety can be estimated by generating different stochastic realizations of the random fields and evaluating the variations of the factor of safety. Under a reliability perspective, a large number of realizations of the stochastic random fields are needed in order to evaluate the probability of failure in a Monte Carlo fashion.

Stationary Gaussian random fields have been generated with a tool developed at the University of Maryland [29] for regular grids using an exponential correlation form of the covariance function.

## 4. Application to a Case Study

### 4.1. Case Study Dam

**Figure 2:**
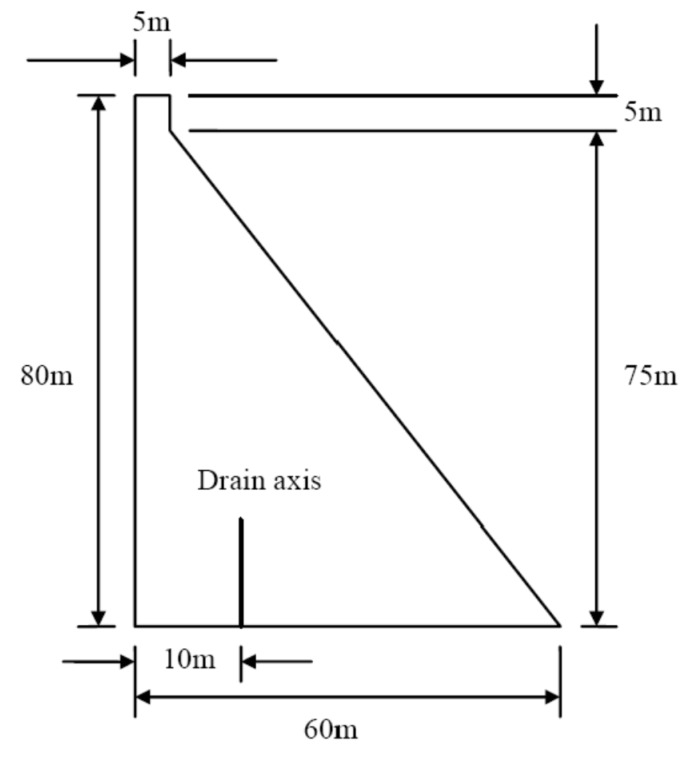
Dam geometry.

We have used the 2D gravity dam model included in Theme C the 11th Benchmark Workshop on Numerical Aspects of Computation on Dams, 2011 [30] as a case study. The dam geometry is defined in Figure 2.

The mass density of the concrete is 2400 kg/m^3^. Data for material properties for dam-foundation contact are given in Table 1. Tensile strength in the contact is assumed to be zero.

The mean value of strength parameters will be kept through the analysis, assuming that the consideration of more or less data only affects the standard deviation and not the mean values. We acknowledge that, in practice, using more data will affect the mean value, but we want to isolate the impact of the increasing number on data on safety levels, as will be shown in following sections.

The considered loadings are self-weight, hydraulic pressure acting on the upstream face of the dam and uplift acting on the base of the dam. Development of a crack at the interface is considered if tensile stresses appear at the contact and uplift distribution is updated accordingly, with full hydrostatic pore pressure along the crack extent. Two cases of drain effectiveness are considered, with discrete probabilities associated: drains fully effective with an uplift coefficient *K* = 0.33 are assumed to have a probability of 0.9 and drains ineffective with an uplift coefficient *K* = 1.00 are assumed to have a probability of 0.1. To simplify the analysis and to be able to compare the different approaches in a simple way only one case of water level in the reservoir is considered. The water level (W.L.), measured over the dam-foundation contact, is 80 m. The probability associated with this level, or annual exceedance probability (AEP) is 10^−4^·year^−1^.

Only two loading combinations are thus analyzed, as defined in Table 2.

**Table 1:** Data for friction coefficient and cohesion at the dam-foundation contact.

Sample	Friction Coefficient	Cohesion (MPa)
1	1.00	0.5
2	0.75	0.3
3	1.03	0.3
4	1.00	0.7
5	1.15	0.8
6	1.33	0.2
7	1.38	0.6
8	1.00	0.0
9	1.15	0.1
10	1.73	0.2
11	1.96	0.2
12	1.88	0.4
13	1.73	0.7
14	1.48	0.1
15	1.88	0.4

**Table 2:** Loading combinations and associated probabilities.

Combination	Water Level (m)	Annual Exceedance Probability (AEP) (year^−1^)	Drains, *K*	*P*rob(K)	Combination Probability (year^−1^)
N°1	80	10^−4^	0.33	0.9	9.00 × 10^−5^
N°2	80	10^−4^	1.00	0.1	1.00 × 10^−5^

The intention of the authors in giving these probabilities is by no means to perform a full probabilistic analysis or to derive conclusions about the total probability of failure of the dam. It is rather to use these numbers to put into context the typical order of magnitude of failure probabilities in dams and to help in the interpretation and contextualization of the impact of the use of different methodological approaches in the failure probabilities.

### 4.2. Approach I. Parameter Average with no Data

In this case, it is assumed that, according to available information of similar dams and geological conditions, the following probability distributions for local strength parameters can be proposed as shown in Table 3: tanφ_local_ ~ N(μ[tanφ_local_] = 1.36; SD[tanφ_local_]) = 0.39; *coh*_local_ ~ N(μ[*coh*_local_] = 0.37 MPa; SD[*coh*_local_] = 0.25 MPa). Both normal distributions have been tested with the Kolmogorov-Smirnov test and the skewness test, and the result is that the null hypothesis: *i.e.*, the sample comes from a population normally distributed, cannot be rejected.

According to the approach followed, the same probability distribution parameters are used for the whole contact plane strength parameters or averaged parameters, as shown in Table 4. The factor of safety (*FS*) is evaluated in two cases. Case (a) uses the 5% fractile of the probability distributions as a cautious estimate of the strength parameter values. The corresponding values are [tanφ_contact_]_5%_ = 0.72 and [*coh*_contact_]_5%_ = 0 MPa. Cohesion is assumed to be null as the 5% fractile corresponds with negative values without physical meaning. Case (b) uses the mean values.

Probabilities of failure are estimated using Monte Carlo simulation. Random values of strength parameters are sampled from their independent, uncorrelated probability distributions, and the sliding stability is calculated for each sample, leading to two possible outcomes: failure or no failure. The probability of failure is estimated as the ratio between the total number of failures and the total number of samples evaluated. The results obtained are shown in Table 5.

**Table 3:** Parameters of normal distributions for local friction coefficient and local cohesion.

μ[tanφ_local_]	SD[tanφ_local_]	μ[*coh*_local_]	SD[*coh*_local_]
1.36	0.39	0.37	0.25

**Table 4:** Parameters of normal distributions for average friction coefficient and average cohesion.

μ[tanφ_contact_]	SD[tanφ_contact_]	μ[*coh*_contact_]	SD[*coh*_contact_]
1.36	0.39	0.37	0.25

**Table 5:** Factor of safety and probability of failure with Approach I.

Combination n°	Water Level (m)	K Drains	*FS* (a)	*FS* (b)	Conditional Probability of Failure	Combination Probability	Probability of Failure
1	80	0.33	1.15	2.53	5.17 × 10^−2^	9.00 × 10^−5^	4.65 × 10^−6^
2	80	1.00	0.69	1.51	3.20 × 10^−1^	1.00 × 10^−5^	3.19 × 10^−6^

Note: Estimate of strength parameters: (a) 5% fractile; (b) mean values.

### 4.3. Approach II. Parameter Average with Variable Amount of Data

To illustrate the effect of increasing the number of data on the safety evaluation, three situations will be considered, corresponding to values of 5, 10 and 15 pairs of values of local friction coefficient and cohesion available. The parameters of normal probability distributions for averaged strength parameters are now obtained by Equations (13)–(16). The corresponding values for *n* = 5, 10 and 15 data are shown in Table 6. The factor of safety is evaluated in two cases. Case (a) uses the 5% fractile of the probability distributions as a cautious estimate of the strength parameter values. The corresponding values are shown in Table 7. Case (b) uses the mean values. The results obtained are shown in Table 8, Table 9 and Table 10.

**Table 6:** Parameters of normal distributions for average friction coefficient and average cohesion.

Number of Data	μ[tanφ_contact_]	SD[tanφ_contact_]	μ[*coh*_contact_]	SD[*coh*_contact_]
-	1.36	0.39	0.37	0.25
5	1.36	0.18	0.37	0.11
10	1.36	0.12	0.37	0.08
15	1.36	0.10	0.37	0.06

**Table 7:** Five percent fractile values for average strength parameters.

Number of Data	[tanφ_contact_]_5%_	[*coh*_contact_]_5%_
-	0.72	0
5	1.08	0.19
10	1.16	0.24
15	1.20	0.26

**Table 8:** Approach II. Results for *n* = 5.

Combination n°	Water Level (m)	*K* Drains	*FS* (a)	*FS* (b)	Conditional Probability of Failure	Combination Probability	Probability of Failure
1	80	0.33	1.84	2.53	3.00 × 10^−4^	9.00 × 10^−5^	2.70 × 10^−8^
2	80	1.00	1.10	1.51	1.58 × 10^−1^	1.00 × 10^−5^	1.58 × 10^−6^

Note: Estimate of strength parameters: (a) 5% fractile; (b) mean values. *FS*: The factor of safety.

**Table 9:** Approach II. Results for *n* = 10.

Combination n°	Water Level (m)	*K* Drains	*FS* (a)	*FS* (b)	Conditional Probability of Failure	Combination Probability	Probability of Failure
1	80	0.33	2.04	2.53	<10^−5^	9.00 × 10^−5^	<9.00 × 10^−10^
2	80	1.00	1.21	1.51	6.93 × 10^−2^	1.00 × 10^−5^	6.93 × 10^−7^

Note: Estimate of strength parameters: (a) 5% fractile; (b) mean values.

**Table 10:** Approach II. Results for *n* = 15.

Combination n°	Water Level (m)	*K* Drains	*FS* (a)	*FS* (b)	Conditional Probability of Failure	Combination Probability	Probability of Failure
1	80	0.33	2.12	2.53	<10^−5^	9.00 × 10^−5^	<9.00 × 10^−10^
2	80	1.00	1.27	1.51	3.85 × 10^−2^	1.00 × 10^−5^	3.85 × 10^−7^

Note: Estimate of strength parameters: (a) 5% fractile; (b) mean values.

### 4.4. Approach III. Parameter Average Using Variance Reduction Factor for Spatial Variability

Now, we want to examine the effect of spatial variability using the concept of the variance reduction factor that considers the effect of the spatial extent of the governing failure mechanism.

We will consider that the scale of fluctuation is the same for both friction coefficient and cohesion. Two possible scales of fluctuation are considered: 10 and 20 m. We will use the formulation of Schneider and Schneider [27], though the results obtained with the formulation proposed by Phoon and Kulhawy [26] are very similar.

The values of the variance reduction factor and total coefficient of variation for friction coefficient and cohesion are shown in Table 11 and Table 12. Parameters of normal distributions for average friction coefficient and average cohesion are shown in Table 13 while 5% fractile values for average strength parameters are shown in Table 14. The results obtained are shown in Table 15 and Table 16.

**Table 11:** Variance reduction factors.

*SOF* (m)	*Lx* (m)	*Ly* (m)	Г*x*^2^	Г*y*^2^	Гs^2^
10	60	15	0.16	0.52	0.08
20	60	15	0.30	0.75	0.22

**Table 12:** Total coefficient of variation including scale of fluctuation and spatial extent of governing mechanism.

*SOF* (m)	Friction Coefficient				Cohesion (MPa)			
	Mean	SD	*COV*	*COV*_TOTAL_	Mean	SD	*COV*	*COV*_TOTAL_
10	1.36	0.39	0.29	0.08	0.37	0.25	0.68	0.19
20	1.36	0.39	0.29	0.14	0.37	0.25	0.68	0.32

**Table 13:** Parameters of normal distributions for average friction coefficient and average cohesion.

*SOF* (m)	µ[tanφ_contact_]	SD[tanφ_contact_]	µ[*coh*_contact_]	SD[*coh*_contact_]
0	1.36	0.39	0.37	0.25
10	1.36	0.11	0.37	0.07
20	1.36	0.19	0.37	0.12

**Table 14:** Five percent fractile values for average strength parameters.

*SOF* (m)	[tanφ_contact_]_5%_	[*coh*_contact_]_5%_
0	0.72	0
10	1.18	0.25
20	1.05	0.17

**Table 15:** Approach III. Results for *SOF* = 10 m.

Combination n°	Water Level (m)	*K* Drains	*FS* Case (a)	*FS* Case (b)	Conditional Probability of Failure	Combination Probability	Probability of Failure
1	80	0.33	2.14	2.53	<10^−5^	9.00 × 10^−5^	<9.00 × 10^−10^
2	80	1.00	1.28	1.51	4.48 × 10^−2^	1.00 × 10^−5^	4.48 × 10^−7^

Note: Estimate of strength parameters: (a) 5% fractile; (b) mean values.

**Table 16:** Approach III. Results for *SOF* = 20 m.

Combination n°	Water Level (m)	*K* Drains	FS Case (a)	FS Case (b)	Conditional Probability of Failure	Combination Probability	Probability of Failure
1	80	0.33	1.81	2.53	7.00 × 10^−4^	9.00 × 10^−5^	6.30 × 10^−8^
2	80	1.00	1.08	1.51	1.64 × 10^−1^	1.00 × 10^−5^	1.64 × 10^−6^

Note: Estimate of strength parameters: (a) 5% fractile; (b) mean values.

### 4.5. Approach IV. Spatially Variable Strength

**Figure 3:**
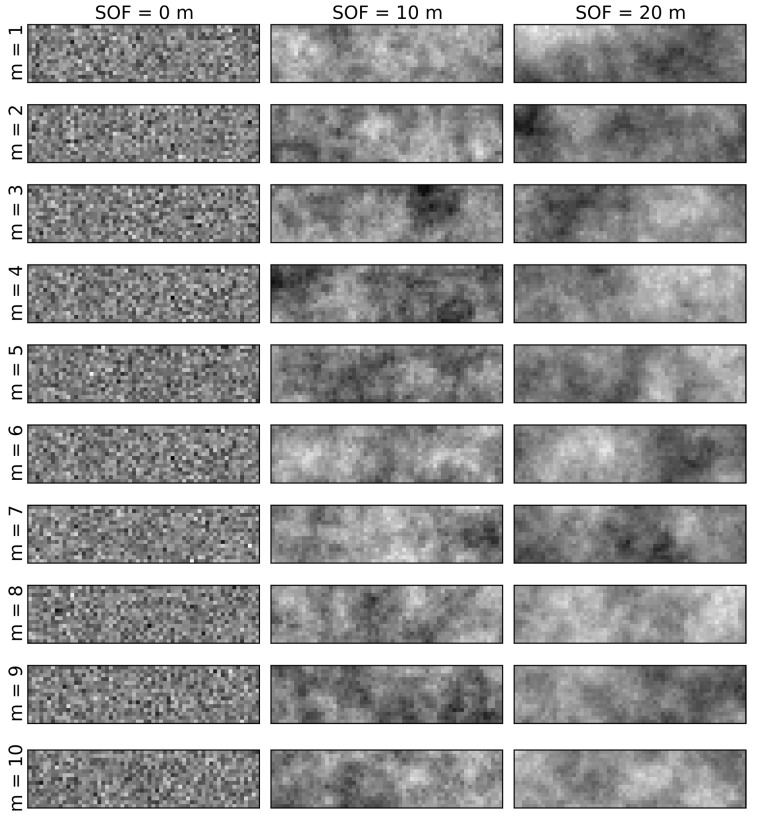
Realization of Gaussian random fields for scales of fluctuation of 0 m, 10 m and 20 m.

Two independent, uncorrelated Gaussian random fields sets are generated, one set for friction coefficient and one set for cohesion. Each random field has an autocorrelation distance of 10 m. The grid is formed by 60 columns and 15 rows. The grid size is 1 m × 1 m. Each set of random fields includes 10 realizations, to evaluate the degree of variation of the factor of safety. Example of random fields generated for different scales of fluctuation are shown in Figure 3.

The stability model used for the dam is a 2D model. For the monolith analyzed, the vertical stress distribution (*Z*-axis) over the dam-foundation contact plane is assumed to vary linearly along the *X*-axis (upstream-downstream) and to be constant along the *Y*-axis (left side-right side). Strength parameters can be averaged along each row (*Y*-axis) for calculations. Sliding strength is calculated using Equation (28). Factors of safety results for spatially varied strength parameters with scale of fluctuation of 0, 10 and 20 m are shown in Table 17, Table 18 and Table 19, respectively.

**Table 17:** Factors of safety results for spatially varied strength parameters with scale of fluctuation of 0 m.

Combination n°	Random Field Realization (*SOF* = 0 m)												
	1	2	3	4	5	6	7	8	9	10	Mean	SD	COV
1	2.60	2.69	2.67	2.65	2.64	2.65	2.67	2.63	2.62	2.61	2.64	0.03	0.01
2	2.04	2.10	2.10	2.08	2.06	2.06	2.09	2.05	2.07	2.04	2.07	0.02	0.01

**Table 18:** Factors of safety results for spatially varied strength parameters with scale of fluctuation of 10 m.

Combination n°	Random Field Realization (*SOF* = 10 m)												
	1	2	3	4	5	6	7	8	9	10	Mean	SD	COV
1	2.85	2.73	2.28	2.56	2.62	3.25	2.77	3.08	3.04	2.44	2.76	0.30	0.11
2	2.31	2.10	1.75	2.16	2.03	2.69	2.14	2.55	2.42	1.83	2.20	0.30	0.14

**Table 19:** Factors of safety results for spatially varied strength parameters with scale of fluctuation of 20 m.

Combination n°	Random Field Realization (*SOF* = 20 m)												
	1	2	3	4	5	6	7	8	9	10	Mean	SD	COV
1	2.65	2.84	2.32	2.10	1.77	2.77	2.63	2.75	2.40	1.45	2.37	0.47	0.20
2	2.04	2.26	1.80	1.54	1.17	2.25	2.17	2.37	1.89	0.95	1.84	0.48	0.26

## 5. Discussion of Results

The results obtained in previous sections are summarized in Table 20 to facilitate comparison. Approaches I and II do not consider explicitly the spatial variability of strength parameters. Approach III focuses on the variability of strength parameters along the failure surface using the variance reduction concept while Approach IV considers the full variability of the shear strength itself, not only parameter variability, along the failure surface. This distinction is important and has practical consequences, as it is shown by the obtained results.

Approach I considers a situation of maximum uncertainty, as the average values of strength parameters for the whole failure surface may take any value from their domain of inherent variability, which is a conservative assumption. It is equivalent to consider an infinite scale of fluctuation. In the absence of enough reliable data, and taking into account the catastrophic consequences associated with a dam failure, it is in line with common practice in the dam safety field. The selection of 5% fractile for characteristic values of strength parameters leads to factor of safety of *FS* = 1.15 for Combination 1 and *FS* = 0.69 for Combination 2, which are the lowest value obtained among the different approaches. The conditional probabilities of failure calculated are consistent with such low factor of safety values, and can be considered as a lower limit in the context of a quantitative risk analysis.

Approach II considers a variance reduction in average strength parameters due to the acquisition of new information. The impact of new data over the dam safety is remarkable, even for low quantity of new data. For *n* = 5 tests on strength parameters the factors of safety are now *FS* = 1.84 for Combination 1 and *FS* = 1.10 for Combination 2. In this latter case, as more information is added to the analysis, the factor of safety increases over the critical value *FS* = 1. For *n* = 15 test on shear strength parameters over a dam-foundation contact, which can be seen as a reasonable upper limit in engineering practice, the factors of safety are almost multiplied by two with respect to those calculated with Approach I, giving *FS* = 2.12 for Combination 1 and *FS* = 1.27 for Combination 2. The conditional probabilities of failure are lowered in a consistent way, as could be expected. For *n* = 5 tests the conditional probability of failure is reduced in two orders of magnitude for Combination 1 and nearly divided by two for Combination 2.

**Table 20:** Factors of safety and probabilities of failure with different approaches.

-		Combination 1				Combination 2			
		*FS* (a)	*FS* (b)	Conditional Probability of Failure	Probabi-lity of Failure	*FS* (a)	*FS* (b)	Conditional Probability of Failure	Probabi-lity of Failure
I: Parameter average (no data)		1.15	2.53	5.17 × 10^−2^	4.65 × 10^−6^	0.69	1.51	3.20 × 10^−1^	3.19 × 10^−6^
II: Parameter average (with N data)	*N* = 5	1.84	2.53	3.00 × 10^−4^	2.70 × 10^−8^	1.10	1.51	1.58 × 10^−1^	1.58 × 10^−6^
	*N* = 10	2.04	2.53	< 10^−5^	< 9 × 10^−10^	1.21	1.51	6.93 × 10^−2^	6.93 × 10^−7^
	*N* = 15	2.12	2.53	< 10^−5^	< 9 × 10^−10^	1.27	1.51	3.85 × 10^−2^	3.85 × 10^−7^
III: Parameter average (var reduction)	*SOF* = 10	2.14	2.53	< 10^−5^	< 9 × 10^−10^	1.28	1.51	4.48 × 10^−2^	4.48 × 10^−7^
	*SOF* = 20	1.81	2.53	7.00 × 10^−4^	6.30 × 10^−8^	1.08	1.51	1.64 × 10^−1^	1.64 × 10^−6^
IV: Spatially variable strength	*SOF* = 0	2.59	2.64	≈0	≈0	2.04	2.07	≈0	≈0
	*SOF* = 10	2.27	2.76	≈0	≈0	1.71	2.20	3.10 × 10^−5^	3.1 × 10^−10^
	*SOF* = 20	1.60	2.37	1.66 × 10^−3^	1.49 × 10^−7^	1.05	1.84	4.06 × 10^−2^	4.06 × 10^−7^

Note: Estimate of strength parameters: (a) 5% fractile; (b) mean values.

The main concern with Approach II is that it ignores the fact that the cohesive and frictional parts of shear strength have different sensibilities to spatial distribution of data from test results. The cohesive part of the shear strength is calculated by multiplying the cohesion results of tests by the area of influence of each test, which can be assumed to be the same for all of them. This means that lower results of cohesion in some tests will be equally compensated by higher results of cohesion in others. On the other hand, this is not true for the frictional part of shear strength. The friction coefficient is multiplied by the effective normal stress, which varies along the failure surface. This asymmetrical normal stress distribution impairs the direct averaging of friction coefficients, as is the product of friction coefficients times the normal stress, *i.e.*, the frictional strength, what should be averaged. Only under the hypothesis that the scale of fluctuation is close to zero, which allows for maximum variability in strength parameters from one point in the contact surface to another point in the vicinity, Approach II will produce meaningful results.

Approach III considers the relationship between the dimensions of the failure surface and the scale of fluctuation of strength parameters, so it is a better, more consistent and faster approach to stability problems with spatial variability, while still using average values for strength parameters. Of course, the difficulty in practical applications is the determination of the scale of fluctuation of strength parameters, or at least a range of feasible scales of fluctuation adequate to perform a sensitivity analysis. The results obtained for the two values of scale of fluctuation analyzed of 10 and 20 m show that for lower scales of fluctuation the safety levels increase while for higher scales of fluctuation the safety levels decrease, with lower factors of safety and higher probabilities of failure. Accepting the results obtained under Approach I as the limit case for an infinite scale of fluctuation, which is a conservative assumption, as the scale of fluctuation decreases the safety levels increase. For *SOF* = ∞ m we have *FS* = 1.15 for Combination 1 and *FS* = 0.69 for Combination 2. For *SOF* = 20 m we get *FS* = 1.81 for Combination 1 and *FS* = 1.08 for Combination 2. This illustrates a useful application of this approach which is to estimate, for a certain problem, what maximum scale of fluctuation would be needed to get *FS* = 1. Further reducing the scale of fluctuation to *SOF* = 10 m increases the safety levels, giving *FS* = 2.14 for Combination 1 and *FS* = 1.28 for Combination 2. In terms of conditional probabilities of failure, a consistent reduction is obtained. For *SOF* = ∞ m we have *P*_cond_ = 5.17 × 10^−2^ for Combination 1 and *FS* = 3.20 × 10^−1^ for Combination 2, while for *SOF* = 20 m we get *P*_cond_ = 7.00 × 10^−4^ for Combination 1 and *P*_cond_ = 1.64 × 10^−1^ for Combination 2 and for *SOF* = 10 m we get *P*_cond_ < 10^−5^ for Combination 1 and *P*_cond_ = 4.48 × 10^−2^ for Combination 2.

Approach IV entails a rigorous consideration of the spatial variability of strength parameters and also the spatial variability of shear strength. This latter aspect is the key difference with Approaches I, II and III. It does not consider probability distributions for average strength parameters, but works with the full probability distribution of local strength values considering their spatial variability using the scale of fluctuation concept. Factors of safety based on characteristic values are no longer calculated. Instead, a set of m factors of safety is calculated using shear strength values obtained from m realizations of random fields for the local coefficient of friction and the local cohesion. Accepting that local cohesion and local friction coefficients are normally distributed, and that the factor of safety is derived as a linear combination of those, it results that the factor of safety is normally distributed as well, allowing estimations of probabilities associated with different factor of safety values.

We have used a short set of *m* = 10 realizations. This number is low for full professional applications but enough to help us to illustrate and compare Approach IV with the rest. Starting with a *SOF* = 20 m, the expected value of the factor of safety is µ[*FS*] = 2.37 and the standard deviation is SD[*FS*] = 0.47 for Combination 1. The range of values obtained is [1.45–2.84] which shows important variability. The estimated conditional probability of failure is *P*rob(*FS* < 1) = 1.66 × 10^−3^. Comparing this results with those obtained for *SOF* = 20 m under Approach III, that give *P*_cond_ = 7.00 × 10^−4^, higher probabilities of failure are obtained with the more rigorous Approach IV, which may indicate that the simplified Approach III may be a little optimistic and does not provide conservative results. On the other hand, for Combination 2, the expected value of the factor of safety is µ[*FS*] = 1.84 and the standard deviation is SD[*FS*] = 0.48. The range of values obtained is [0.95–2.37] which shows important variability as well. The estimated conditional probability of failure is *P*rob(*FS* < 1) = 4.06 × 10^−2^. Comparing again this results with those obtained with Approach III, *P*_cond_ = 1.64 × 10^−1^, slightly lower probabilities of failure are obtained with the more rigorous Approach IV. The former comparison shows that no clear conclusions can be drawn using Approach III as it may under or over-estimate probabilities of failure.

If we consider a smaller scale of fluctuation, *SOF* = 10 m, we have the following results. The expected value of the factor of safety is µ[*FS*] = 2.76 and the standard deviation is SD[*FS*] = 0.30 for Combination 1. The range of values obtained is [2.28–3.25] which shows important variability. The estimated conditional probability of failure is *P*rob(*FS* < 1) = 3.00 × 10^−9^ which is probably lower than the figure obtained with Approach III, that gives *P*_cond_ < 10^−5^. For Combination 2, the expected value of the factor of safety is µ[*FS*] = 2.20 and the standard deviation is SD[*FS*] = 0.30. The range of values obtained is [1.75–2.69]. The estimated conditional probability of failure is *P*rob(*FS* < 1) = 3.10 × 10^−5^, which is lower than the value of 4.48 × 10^−2^ obtained with Approach III.

As the scale of fluctuation is further reduced, the safety level is increased. For a limit case of *SOF* = 0 m, which represents a Gaussian random field with no autocorrelation, the following results are obtained. The expected value of the factor of safety is µ[*FS*] = 2.64 and the standard deviation is SD[*FS*] = 0.03 for Combination 1. The range of values obtained is [2.60–2.69] which shows very low variability. The estimated conditional probability of failure is *P*rob(*FS* < 1) = 0. For Combination 2, the expected value of the factor of safety is µ[*FS*] = 2.07 and the standard deviation is SD[*FS*] = 0.02. The range of values obtained is [2.04–2.10]. The estimated conditional probability of failure is *P*rob(*FS* < 1) = 0.

It is concluded that the key impact of considering a reduced scale of fluctuation is not the variation in the absolute value of the factor of safety, but the reduction on its variability, which leads to very low to null probabilities of failure in practice. The paradox is that *FS* exhibit a not monotonic variation but the probability of failure shows a monotonic increase with *SOF*. This fact shows how relevant can the assessment of a scale of fluctuation be, or at least the assessment of a range of feasible values for the scale of fluctuation, of the spatial variability of strength parameters in the context of a quantitative risk analysis applied to a dam or a similar civil infrastructure.

**Figure 4:**
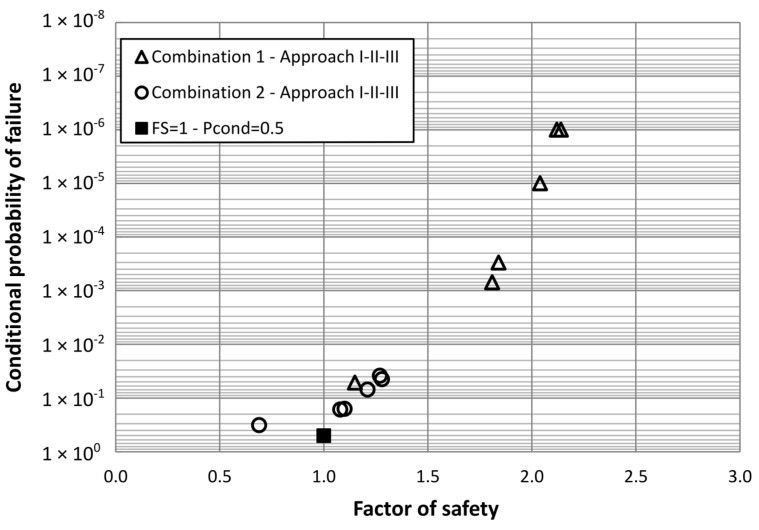
Factor of safety *vs.* conditional probability of failure using 5% fractile for strength characteristic values.

Another aspect of interest derived from the analysis performed is the relationship between factor of safety and conditional probability of failure. Plotting the factor of safety values obtained for Approaches I, II and III using the (a) definition, which considers 5% fractile for characteristic values of strength parameters, *versus* conditional probability of failure we get Figure 4. In this Figure, a singular point of reference which corresponds to (*FS* = 1; *P*_cond_ = 0.5) has been included. It is important to stress the fact that the shown probabilities are conditional probabilities, not total probabilities. This Figure shows the ability of the factor of safety to capture the conditional probability of failure in a consistent way, for different hypothesis of available information and scale of fluctuations of the strength parameters, thus providing a reference to interpret the probabilistic results of a risk analysis applied to a dam. The authors have been using similar plots to help dam engineers not familiar with risk concepts in the interpretation of risk analysis.

**Figure 5:**
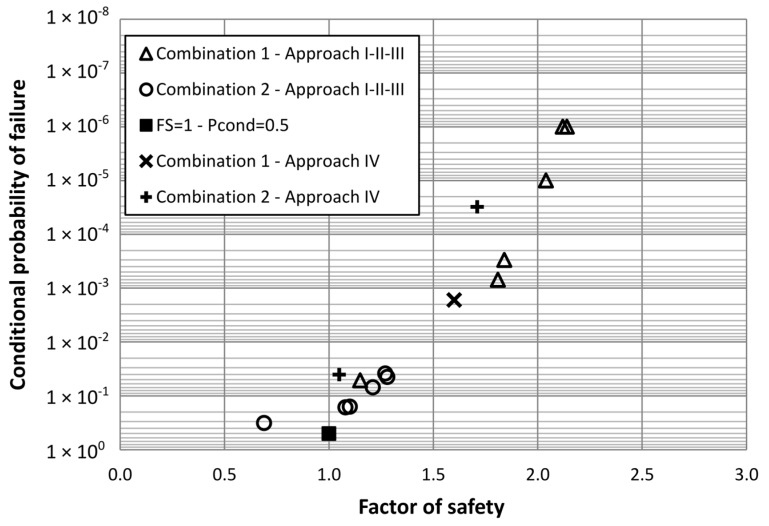
Factor of safety *vs.* Conditional probability of failure using 5% fractile for strength characteristic values, and including 5% fractile of factor of safety distribution for Approach IV.

We have deliberately omitted the plotting of the factors of safety and conditional probability of failure values obtained for Approach IV, as the definition of the factor of safety in this case is different and no comparison can be made directly. To be able to compare similar factors of safety, we have plotted in Figure 5 the values of the conditional probability of failure for Approach IV, which is the *P*rob(*FS* < 1) *versus* the 5% fractile of the factor of safety distribution obtained, assuming normal probability distribution. It can be seen that the points align well with the rest of pairs of values calculated with other approaches.

## 6. Conclusions

Back in 2011, Christian and Baecher [31] suggested a short list of 10 unresolved questions in geotechnical risk and reliability analyses that ranged from technical problems to matters of communication, with the purpose of challenging the reliability community. The first three questions were:

1. Why are failures less frequent than predicted? Typical coefficients of variation for soil engineering properties are reported to be on the order of 20%–30%. Presuming a mean factor of safety of 1.5, corresponding reliability indices (β) are about 1.67, implying probabilities of failure of about 0.05. These are an order of magnitude larger than the observed frequency of adverse performance.
2. What is the actual variability of soil and rock properties? Variations in soil engineering data involve at least: (1) actual variability from one point to another; and (2) noise. In addition, there are at least two bias errors that creep into assessments: (3) statistical error due to limited number of observations; and (4) model error due to the approximate nature of our mathematical descriptions of soil behaviour.
3. What are the effects of spatial correlation? Geological materials arrive at their present configurations by a geologic process that follows physical principles. Therefore, their physical properties exhibit spatial correlation. While there have been successes in describing spatial correlation statistically and in modelling spatially correlated variables, the techniques for dealing with spatial correlation are difficult to implement, they are poorly understood in practice, thus their consequences are often ignored.

In this paper we have addressed some aspects closely related with these three questions. We have shown a strategy to analyze the sliding along the dam-foundation contact for concrete gravity dams under a reliability-based approach, considering explicitly the spatial variability of strength parameters, and comparing the results obtained with typical approaches followed in common practice in the dam safety field. The methodology shows how to evaluate the impact of the scale of fluctuation of strength parameters on safety levels in terms of factors of safety and probability of failure and we have also shown how significant this impact can be.

Data in Table 1 do not come from any real specific dam, but we have prepared them for the exercise performed in the referenced workshop on the basis of values observed at different sites. The advantage of using those values is that they have been already handled by several research teams on a formal exercise. We felt that this was more adequate than producing a set of invented values or using a set of few real-dam values, as we wanted to demonstrate the impact of the number of data on the safety evaluation. If real data were used, one could be prone to assign the particular value obtained in that part of the contact to an area in particular. Our paper demonstrates the need to have an estimate of the order of magnitude of the scale of fluctuation before doing this exercise, as results may be misleading.

This kind of analysis can be incorporated in risk-informed dam safety reviews to assess the safety level, helping in the identification of best actions related to the acquisition of new data and if there is a justification for more elaborated models. During the risk assessment, the need for additional or more refined deterministic or probabilistic analyses could become evident. Ultimately, the engineer needs to combine both traditional engineering analyses and risk assessments to obtain a good understanding of the structure’s expected performance and risk.
